# A framework to support risk assessment in hospitals

**DOI:** 10.1093/intqhc/mzy194

**Published:** 2018-09-01

**Authors:** Gulsum Kubra Kaya, James R Ward, P John Clarkson

**Affiliations:** Engineering Design Centre, Department of Engineering, University of Cambridge, Trumpington Street, Cambridge, UK

**Keywords:** risk assessment, design for safety, patient safety, risk management, guidelines

## Abstract

**Quality problem or issue:**

A number of challenges have been identified with current risk assessment practice in hospitals, including: a lack of consultation with a sufficiently wide group of stakeholders; a lack of consistency and transparency; and insufficient risk assessment guidance. Consequently, risk assessment may not be fully effective as a means to ensure safety.

**Initial assessment:**

We used a V system developmental model, in conjunction with mixed methods, including interviews and document analysis to identify user needs and requirements.

**Choice of solution:**

One way to address current challenges is through providing good guidance on the fundamental aspects of risk assessment. We designed a risk assessment framework, comprising: a risk assessment model that depicts the main risk assessment steps; risk assessment explanation cards that provide prompts to help apply each step; and a risk assessment form that helps to systematize the risk assessment and document the findings.

**Implementation:**

We conducted multiple group discussions to pilot the framework through the use of a representative scenario and used our findings for the user evaluation.

**Evaluation:**

User evaluation was conducted with 10 participants through interviews and showed promising results.

**Lessons learned:**

While the framework was recommended for use in practice, it was also proposed that it be adopted as a training tool. With its use in risk assessment, we anticipate that risk assessments would lead to more effective decisions being made and more appropriate actions being taken to minimize risks. Consequently, the quality and safety of care delivered could be improved.

## Introduction

Across the world, healthcare has devoted substantial attention to ensuring safety [1–4]. A number of studies have been published, such as in relation to safety culture [4] and the reduction of harm [5–8]. Through continuing efforts to improve safety, reforms have been proposed that have been driven by safety–critical industries (e.g. nuclear and aviation), such as the implementation of risk management system [9]. So far, however, such reforms have largely prioritized the investigation of incidents over their prevention in the first place [3]. An approach which focuses on risk assessment [10–12] could complement this reactive practice. Risk assessment, as a part of the overarching process of risk management, aims to identify, analyse and evaluate risks that may have a negative influence on the quality and safety of the care delivered [11, 13–16].

In the National Health Service in England (NHS England), hospitals assess a range of risks, including wrong medication, delayed discharge, patient claims and failure to comply with requirements. In so doing, hospitals provide risk assessment guidelines and training to support their staff—often frontline and risk management staff—and external authorities support and investigate hospitals to deliver safe care [15, 17–19]. However, despite considerable efforts being made, a number of problems have been identified with current practice. For instance, patient safety-related risks can be ignored at the organizational level, and health information technology innovations can be assumed to be safe until something goes wrong [20]; risk assessment techniques are little-used, and if used, they may be used without training [12, 21]; risk register systems can be used as bureaucratic data collection rather than to diagnose potential problems [19]; there can be a lack of consultation with a sufficiently wide group of stakeholders including patients [22]; risk assessment practice is criticized as lacking in consistency and transparency [17]; and the risk evaluation guidance provided is insufficient, which may lead to poor decisions being made [15]. Consequently, risk assessment may be underutilized when attempts are made to ensure safety.

Many of the problems stem from the foundations of risk assessment, including how to express risk, how to analyse it and how to use risk assessment as a tool to improve patient safety [14]. One way to address such problems is through providing good guidance on the fundamental aspects of risk assessment [10, 23]. This paper, therefore, reports the design process for—and content of—a risk assessment framework (RAF). The RAF aims to guide healthcare staff on risk assessment as well as to address current challenges by learning from prescribed good risk assessment practice and the experience of healthcare staff (e.g. doctors, nurses and managers).

## Study design

**Figure 1:**
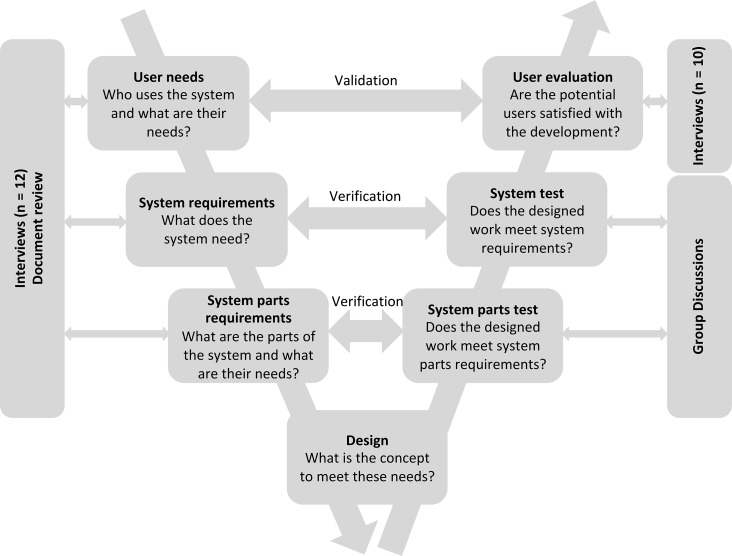
V system developmental model applied for the design of the RAF.

This study adopted a V system developmental model [24, 25] (see Fig. 1) to design the proposed RAF through the consideration of user needs, requirements, multiple design concepts and the evaluation of the selected concept.

Semi-structured interviews were conducted with healthcare staff from different professions in multiple acute hospitals in NHS England to understand user needs in risk assessment (see Table 1). A purposive sampling strategy was used to ensure participants had sufficient experience of risk assessment. All potential participants were known to the research team, and we received permission to interview 12 individuals. The inclusion criterion was to select participants who had been involved in at least one risk assessment. Interview questions were developed based on the literature findings, with further input from the research team and were then piloted with a healthcare researcher. Participants were asked questions in relation to their understanding of risk assessment (e.g. why and how to assess risks), their practical experience in risk assessment (e.g. which methods to use and how to prioritize risks), their recommendations on how to improve risk assessment practice (e.g. participants’ views on the terminology and methods used), their views on their own organizational risk assessment guidelines, their views on good risk assessment practice (e.g. the accessibility and usability of the guidelines they use) and the challenges that they experience when undertaking risk assessment.

**Table 1:** The characteristics of the participants in the user needs interview

Identifier	Type of trust	Job title	Experience in NHS (years)	Safety management training
T1	Acute	Head of integrated clinical governance	38	Risk assessment, risk management, FMEA and RCA
T2	Mental health	Team leader	27	Risk assessment and suicide prevention
F1	Acute teaching	An anaesthetist	9	Simulator training
T3	Other	Head of patient safety investigation	33	Risk management
T4	Acute	Clinical engineer	7	Managing safely, RCA and risk management
T5	Acute	Clinical engineer	10	Risk assessment
T6	Mental health	Team leader	15	Risk assessment and risk management
T7	Mental health	Patient safety practitioner	15	Risk assessment
T8	Other	Risk management consultant	10	Health and safety risk assessment
T9	Acute teaching	Quality improvement fellow	16	Risk management
F2	Acute specialist	Head of nursing	30	Risk assessment, RCA and risk management
T10	Acute specialist	Risk manager	30	Health and safety, risk management, RCA, IOSH, risk officer and human factors

T, telephone interviews; F, face-to-face interviews.

Requirements for the new RAF were elicited from the interviews, an extensive literature review and analysis of risk assessment-related documents from 100 hospitals and 35 risk assessment standards from other industries. The findings were reviewed by the authors to ensure that there were no conflicting or inconsistent requirements. From this, the authors identified 23 requirements to design the RAF (see Table 2).

**Table 2:** Requirements for the design of the RAF

Requirement description	Requirement sources		
	Standards	Policies and procedures	Interviews
1. The system should be described prior to the assessment	[40–42]	x	
2. A comprehensive list of risk sources should be considered when identifying risks	[27, 29, 43]	x	
3. Both known and unforeseen risks should be sought	[44]		
4. An event should be identified by considering objectives and links with other events	[43, 45, 46]	x	x
5. Contributory factors to events should be identified	[47, 48]		
6. Consequences should be identified by considering all impact domains in line with both immediate and knock-on effects	[40, 43, 49]	x	
7. Risks should be properly categorized to help the management of all risks	[10]	x	x
8. All existing controls should be determined to estimate the real level of risk	[40, 43, 50–53]		
9. Risk scores should not be the sole basis on which to make risk-based decisions	[40, 43]		x
10. Uncertainties should be determined when assessing risks	[54]		
11. Tolerability of a risk should be determined based on risk level, codes of practice and comparison with similar reference system(s)	[46]		
12. Risks can be prioritized by consideration of risk levels in combination with other factors	[55, 56]		x
13. Eliminative, detective and reductive control actions should be listed	[53]	x	
14. Risk assessment should be implemented utilizing assessment methods as well as communication and consultation at all times	[40, 43, 57]		
15. Risks should be documented, findings should be shared and risks should be monitored	[40, 50]	x	x
16. Ordinary language should be used in risk assessment			x
17. The improved approach should fit on an A4 sheet			x
18. The framework should support a quick risk assessment			x
19. Risk assessment should be systematic		x	x
20. The framework should be easy to use			x
21. The framework should be adaptable to all contexts and should guide the assessment of all types of risks			x
22. The framework should be easily accessible when required			x
23. The framework should be compatible with other risk assessments tools and methods			x

Using the refined list of requirements, through multiple discussions the authors developed a range of design concepts, which were finally refined into a single concept. The authors then evaluated the framework through the use of a scenario as follows:

In a hospital setting, a neurorehabilitation unit will be moved from an old building to a new building, and the standards of the patient rooms will be changed. Since there is a change in the system, a risk assessment will be conducted to assess risks in the new neurorehabilitation unit before the move occurs. As a part of this, a risk assessment will be conducted to assess all risks in relation to the patient’s accommodation in a single-bed patient room.

User evaluation was conducted with 10 participants (8 were new participants) (see Table 3). The evaluation interviews comprised two parts. In the first part, the author (G.K.K.) explained the how the RAF could work with the risk assessment scenario given above. In the second part, each participant discussed their views on the 17 predetermined statements. A Likert scale was then used by the participants to rate their level of their agreement to each statement. The average rating was calculated by assigning a score to each Likert scale (i.e. ‘strongly agree = 5,’ ‘agree = 4, neutral = 3,’ ‘disagree = 2’ and ‘strongly disagree = 1’) in order to aid numerical analysis. In addition to the responses of the user evaluation statements, participants provided brief comments on two open-ended questions to improve the initial version of the framework: ‘what is familiar and what is new about the RAF?’ and ‘what changes would you recommend to improve the RAF?’ and were given additional space to add further comments.

**Table 3:** The characteristics of the interview participants for the evaluation

Identifier	Type of trust	Job title	Experience in NHS (years)	Frequency of involvement in a risk assessment
I1^a^	Acute teaching 1	Anaesthetist	9	Rarely
I2	Acute teaching 1	Clinical scientist	15	Weekly
I3	Acute specialist 1	Head of risk management	15	Daily
I4	Acute specialist 1	Head of governance and improvement	8	Rarely
I5	Acute teaching 1	Clinical engineer	15	Bimonthly
I6	Acute teaching 2	Corporate risk manager	8	Weekly
I7^a^	Acute specialist 2	Risk manager	34	Daily
I8	Other	Consultant in risk leadership	25	A few times in a week
I9	Acute teaching 3	Quality and safety manager	10	Daily
I10	Acute teaching 4	Clinical director	35	Monthly

^a^Participants who had also been involved in the previous interview process.

The design of the RAF was then developed iteratively to improve its usefulness and usability based on the comments given and discussions between the authors.

## Risk assessment framework

Having considered all requirements, an RAF was designed by the authors, consisting of a risk assessment model, explanation cards and a risk assessment form.

**Figure 2:**
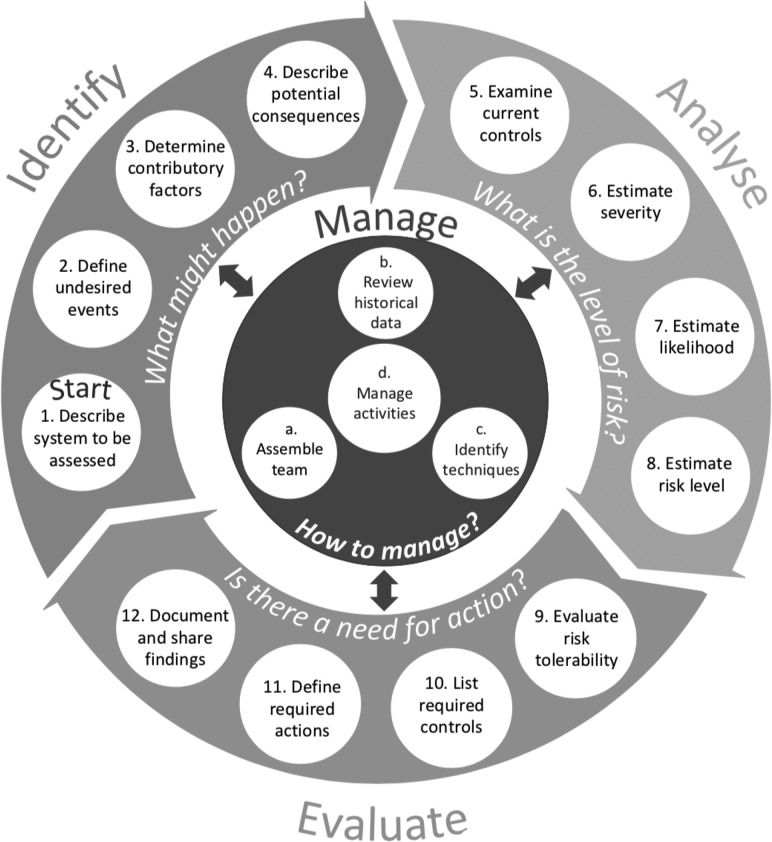
Risk assessment model.

The risk assessment model comprises four phases (identify, analyse, evaluate and manage), and each phase comprises four steps (see Fig. 2).

**Figure 3:**
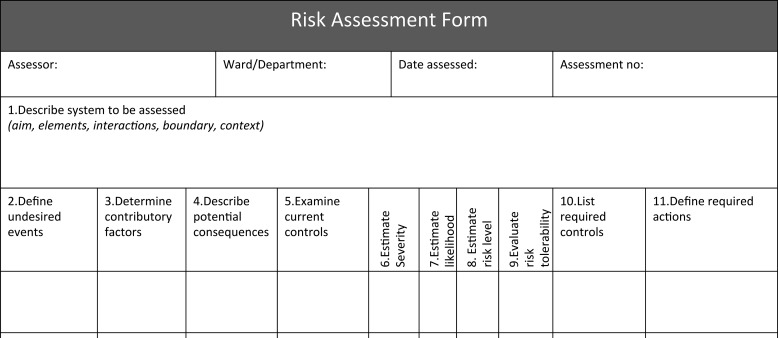
Risk assessment form.

Each step is described on an A5 size double-sided explanation card (see Appendix 1), which provides a number of prompts. Table 4 summarizes these prompts at each risk assessment step. These cards are designed to assist healthcare staff in undertaking risk assessment since they bring together the key principles of risk assessment. The risk assessment form is provided to document the risk assessment findings (see Fig. 3).

**Table 4:** A brief summary of the prompts provided for each assessment step

Assessment Step [Question to be responded to]	Prompts to be considered
**Identify—‘what might happen?’**	
1. Describe system to be assessed ‘What is being assessed and how does the system work?’	− Assessment aim − System boundary − System elements and their interactions − System context
2. Define undesired events ‘What could go wrong?’	− System description − Extreme cases − Undesired event categories (e.g. clinical and organizational)
3. Determine contributory factors ‘What could contribute to the occurrence of undesired events?’	− Patient − Staff − Task − Communication − Equipment − Control actions − Organizational − Environmental
4. Describe potential consequences ‘What are the potential consequences of the undesired events?’	− Impacts on people (e.g. harm) − Impacts on organization (e.g. staffing and claims) − Impacts on environment (e.g. hospital waste) − Immediate effects − Knock-on effects
**Analyse—‘what is the level of risk?’**	
5. Examine current controls ‘What are the current controls and how effective are they?’	− Controls to prevent undesired events − Controls to detect undesired events − Controls to reduce the severity of the consequences − The level of effectiveness of these controls
6. Estimate severity ‘How severe are the described risks?’	− A rating scheme − Consequence descriptions of each rating for each impact area
7. Estimate likelihood ‘What is the likelihood of occurrence of the consequences?’	− A rating scheme − Frequency descriptions to be used for continuous operations − Probability descriptions to be used for one-off projects
8. Estimate risk level ‘What is the level of risk?’	− A combination of the likelihood and consequence of a risk (e.g. quantitatively or qualitatively)
**Evaluate—‘is there any need for action?’**	
9. Evaluate risk tolerability ‘How tolerable is the risk?’	− Risk level (e.g. low risks are generally tolerable and high risks are generally intolerable) − Written rules (e.g. standards and legal requirements) − Potential benefits of taking the risk
10. List required controls ‘What new controls are required to modify the risk?’	− Ineffective existing controls − Contributory factors − Controls to prevent undesired events − Controls to detect undesired events − Controls to reduce the severity of consequences
11. Define required actions ‘What actions are required to implement the new controls?’	− Creating a list of required actions − Action prioritization by considering the criticality of the risks − Management responsibility for these actions − Review frequency
12. Document and share findings ‘What are the findings and what lessons are learnt?’	− System description − Limitations and assumptions made in the assessement − Assessment methodology − Risk assessment findings and results − Discussions of the results − References
**Manage—‘how to manage?’**	
a. Assemble team *‘*Who should be in the assessment team?’	− A facilitator who has experience in risk assessment − A multidisciplinary group of experts in the system to be assessed
b. Review historical data ‘What can be learnt from historical data?’	− Incident reports − Patient complaints and claims − Quality and performance reports − Safety alerts − Audit reports − Reports from external authorities − Academic literature
c. Identify technqiues ‘Which techniques should be used?’	− System diagrams or flow charts for system description − Peer review and team discussions to improve judgement − Brainstorming, SWIFT and the Delphi technique to identifiy all risks − Bow-tie analysis to display the pathway of an event and to examine curent controls − FMEA to identify the ways failure could occur and the way they could be treated − Risk matrices to help determine risk tolerability and to allocate resources − Specific risk assessment forms (e.g. patient falls and moving and handling)
d. Manage activities ‘How should people, data and techniques be deployed throughout risk assessment?’	− Coordination of all risk assessment activities − Communication and consultation with all stakeholders of the assessment at all times − Iterating through all steps of the risk assessment model − Monitoring and reviewing assessed risks on a regular basis as well as when there is a change − Tailoring the framework to fit assessment needs

In the identify phase, the system is described, potential undesired events are defined, their contributory factors are determined and their potential consequences are identified. This phase seeks to answer the question: ‘what might happen?’.

In the analyse phase, current controls are examined, and the severity of the consequence, the likelihood of occurrence and the level of risk are estimated. This step aims to address the question: ‘what is the level of risk?’.

In the evaluate phase, the estimated risk level is compared with the risk criteria (e.g. up to a risk score of 9 is generally tolerable) to decide whether or not the risk is tolerable and if there is any need to take any action. Any controls required are listed, and findings of the assessment are documented as well as shared. This phase, therefore, aims to address the question: ‘is there any need for action?’.

In the manage phase, which interacts with all the phases of risk assessment, a team is assembled, historical data are reviewed, techniques to be used in risk assessment are identified, and all activities related to these should be managed. Thus, this phase coordinates the management of all steps to conduct effective risk assessment by seeking to answer the question: ‘how to manage?’.

## Evaluation of the RAF

The authors evaluated the framework by conducting a risk assessment through the use of the predetermined scenario. The authors identified 20 potential undesired events (e.g. patient falls); described a wide range of contributory factors (e.g. staff tiredness) and multiple consequences for each potential undesired event (e.g. treatment delay); examined current controls; estimated risk levels; evaluated their tolerability; listed required controls (e.g. replacement of the bed rails) and defined actions associated with the controls (e.g. responsibilities for implementation). This allowed the authors to crudely evaluate the usefulness of the framework, and, therefore, to develop the initial version of the framework iteratively. For instance, the initial version of the framework encouraged risk sources to be identified first, followed by risk scenarios. Subsequently, this was reversed, with the identification of undesired events coming first and then contributory factors, which are considered as risk sources. This was due to the fact that it was easier to identify the undesired event first. Indeed, undesired events are often known in healthcare [20] and it is more challenging to identify risk sources [10].

Each interview for the user evaluation lasted approximately 80 min. Results are shown in Table 5.

**Table 5:** Results from the user evaluation statements (RAF = risk assessment framework)

Statements	Strongly agree	Agree	Neutral	Disagree	Strongly disagree	Average
Usefulness						
I would be likely to identify more risks by using the RAF	2	3	4	1		3.6
I would be likely to analyse risks more effectively by using the RAF		9	1			3.9
I would be likely to better evaluate risks by using the RAF	1	8	1			4
I would be likely to assess risks more systematically by using the RAF	4	3	3			4.1
I found the RAF useful to guide me on risk assessment	3	6	1			4.2
Using the RAF could make me more confident about risk assessment	2	4	4			3.8
Using the RAF could improve current risk assessment practice	5	4	1			4.4
Using the RAF could make patients safer	3	2	4	1		3.7
Perceived usability						
I found the RAF clear and understandable	3	6	1			4.2
I found the RAF easy to use	2	7	1			4.1
I found the RAF easily compatible to our existing approach	2	7	1			4.1
Expected value						
The RAF improved my current knowledge on risk assessment	1	3	4	2		3.3
The RAF increased my awareness on risk assessment	1	1	6	2		3.1
The RAF could be beneficial to guide me on risk assessment	4	3	2	1		4
I can see the value in having the RAF	6	2	2			4.4
It is worth spending more time on risk assessment to use the RAF	3	4	3			4
Switching from the old approach to the RAF is essential	3	3	3		1	3.7
What is familiar and what is new about the RAF?						
What changes would you recommend to improve the RAF?						

RAF, risk assessment framework.

Regarding the open-ended questions, all participants responded to the first question. For example, I10 stated, ‘The general framework is familiar. However, it builds in a more robust and comprehensive approach to risk assessment and risk control.’ I5 highlighted the reduced jargon and technical terms and found it very useful. Similarly, I8 pointed out that ‘the methodology is presented in much more user-friendly terms than by experts such as ISO 31 000 and the Health and Safety Executive*.*’ I9 did not find the RAF to be significantly different to current standards, but highlighted that the inclusion of the contributory factors was the part she found the most useful. She also added ‘I would see my primary use of the RAF as a training aid used during face to face training sessions, with staff then able to use the RAF as a post-training prompt to remind them of the steps they need to follow when carrying out a risk assessment.’ Other participants also claimed that the RAF is familiar to them in terms of its main steps (i.e. identify, analyse and evaluate), but they found the details to be different, systematic and helpful.

Seven of the participants responded to the second question. I9 provided three recommendations: to have stronger linkages between contributory factors and controls/actions, to consider estimating a target risk score as well as the actual risk score and to clarify to what extent to follow the RAF and when to do so. I8 recommended stronger links to objectives and that the framework should allow opportunities to be assessed as well as downside risks. I10 suggested there should be more explanation of what ‘system’ means. I7 suggested adding technique cards to explain a number of techniques to support risk assessment, whereas I4 claimed that adding such cards would make it too complicated. I2, I3 and I6 recommended developing specific cards for different users and use-cases (such as medical devices, and clinical and organizational risk assessments).

Seven participants provided further comments. I7 stated that the RAF closely follows their new risk management training handbook. I4 found the framework to be well presented and simple to understand and stated that it could be used as a teaching aid. I2 found it very accessible and easy to follow. I5 stated that they would like to implement it, I6 found it to be a useful tool, and I1 found the team approach necessary and found the RAF to be ‘really good’. I10 also appreciated the work by stating, ‘I think this is an excellent framework that will help many people’.

Based on the findings from the interviews, the authors further developed the initial design by providing additional guidance on the estimation of likelihood and consequence; developing a ‘abridged’ version of the explanation cards; colouring each phase with a different colour and numbering each card.

## Discussion

This study presented an RAF to guide healthcare staff in undertaking risk assessment. The framework was designed to be systematic and compatible with existing risk assessment practice. In essence, the framework simplifies the risk terminology used, and it brings together the principles of national and international risk assessment standards as well as a number of techniques (e.g. failure mode and effects analysis (FMEA), root cause analysis (RCA) and bow-ties). However, the framework should be tailored to the specific needs of the assessment. For example, local hospital requirements might require a slightly modified set of risk criteria or the use of a more specific contributory factors list. Additionally, we believe the framework could readily be used in different healthcare settings (e.g. primary care), in the UK and worldwide, since it provides guidance on the fundamental aspects of risk assessment.

The framework has the potential to identify a large number of risks through consideration of the system to be assessed, its parts and their interactions, and it has the potential to determine a wide range of contributory factors. In healthcare, contributory factors are often determined following an incident by the use of RCA. Yet, too often only a single cause is identified [26] despite the provision in the healthcare literature of a number of lists of multiple potential contributory factors [27–29]. Identifying many contributory factors not only helps to understand potential undesired events but also helps to set up effective controls in the system in order to prevent, detect or reduce the severity of the undesired events. However, it should also be noted that identifying many risks does not necessarily lead to better risk controls [30]. Even a good risk assessment does not lead to safe systems if the findings of the risk assessment are not implemented.

Furthermore, the framework suggests determining the tolerability of a risk through the consideration of multiple factors, including risk scores, organizational and regulatory requirements and the potential benefits of leaving the risk in place, and the framework urges its potential users to assemble a multidisciplinary team to undertake risk assessments. In the English NHS, organization-wide risks tend to be evaluated by individuals through the use of risk matrices in which consequence and likelihood axes are used and categorized, each with a score from 1 to 5 [15]. However, the use of risk matrices, and thus risk scores, has been criticized in the literature. To gain (or avoid) attention, lower risk scores can be artificially recategorized to a higher risk level (or vice versa), and risk scoring itself can be subjective [15, 31, 32] and can lead to biased judgements about the management of the risks [33, 34]. However, taking into account multiple factors to determine the tolerability of a risk would help minimize the limitations of the use of risk matrices, and the involvement of a multidisciplinary team would help to minimize subjectivity in risk scoring.

While the RAF has been shown to offer great value in supporting effective risk assessment, there are some limitations to this study. First, the list of requirements is not intended to be exhaustive and constitutes only one approach to addressing the problems identified in this paper.

Second, the framework is built on the Safety I approach, whereas the Safety II approach might make a significant contribution to the current risk assessment practice, by considering success rather than only undesired events [35, 36]. However, this approach has not yet been widely used and there are a number of criticisms regarding its usability and adoptability [37–39].

Third, the evaluation of the RAF was limited. The evaluation interviews were not designed to provide duplicating and reversing questions to establish the reliability of the responses. Additionally, participants’ views on the proposed RAF could have been biased since they evaluated the framework based on the explanations presented rather than on their experience of using it. While the authors of this paper aimed to minimize such limitations by conducting regular review meetings, a control study could be conducted to see the real impact of the framework in comparison to typical current risk assessment practice in hospitals.

## Conclusion

Risk assessment supports decisions made in relation to potential undesired events. Despite significant efforts of healthcare professionals and organizations, there is a potential to improve current risk assessment practice in hospitals. In this paper, we designed an RAF to guide healthcare staff in risk assessment and to address existing challenges of risk assessment. This was subsequently evaluated to investigate its practical use.

The framework brings the principles of risk assessment together and learns from the experience of healthcare staff in risk assessment. It uses simplified risk terminology to minimize misconceptions and encourages: convening a multidisciplinary team, describing the system to be assessed, defining potential undesired events based on the system description, determining a wide range of contributory factors, considering all potential consequences, determining tolerability of a risks by considering multiple factors and considering control actions to minimize the potential undesired events defined.

The framework can be used as a training tool to guide in effective risk assessment as well as a tool to assess risks in healthcare settings. We believe that the framework can contribute to the quality and safety of care when it is used effectively and the assessment findings are implemented.

## Supplementary Material

Supplementary Data

Supplementary Data
