# Supplementary material for: A framework to support risk assessment in hospitals
Source: Int J Qual Health Care. 2018 Sep 1;31(5):393–401. doi: 10.1093/intqhc/mzy194 (PMC6528703; doi:10.1093/intqhc/mzy194)
Supplement: Supplementary Data [file mzy194_appendix_1_risk_assessment_explanation_cards.docx]

Appendix 1 Risk assessment explanation cards
