# Supplementary material for: A framework to support risk assessment in hospitals
Source: Int J Qual Health Care. 2018 Sep 1;31(5):393–401. doi: 10.1093/intqhc/mzy194 (PMC6528703; doi:10.1093/intqhc/mzy194)
Supplement: Supplementary Data [file mzy194_raf_final_cards_bw.pdf]

Risk Assessment Framework

Identify | Analyse | Evaluate | Manage

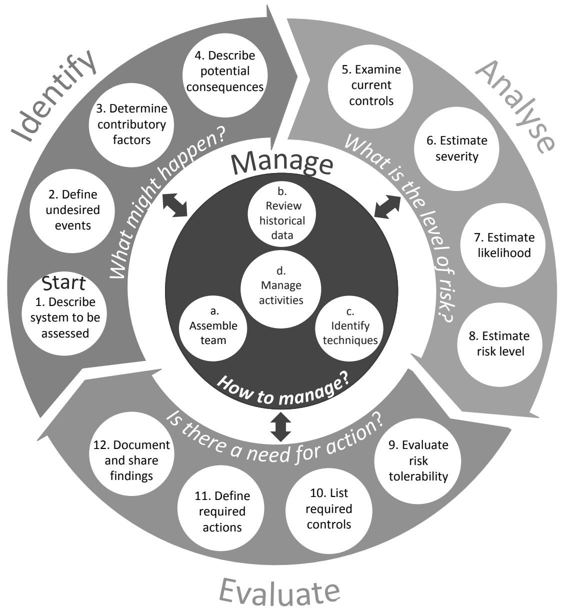

Risk Assessment Framework

This framework provides guidance on risk assessment. It contains four steps: *Identify*, *Analyse*, *Evaluate* and *Manage* steps.

−*Identify* addresses the question of "*What might happen?*"

−*Analyse* addresses the question of "*What is the level of risk?*"

−*Evaluate* addresses the question of *"Is there a need for action?"*

−*Manage* supports the management of all steps

This framework is intended as a guide. It can be tailored to fit assessment needs

GKK

Risk Assessment Framework

Identify | Analyse | Evaluate | Manage

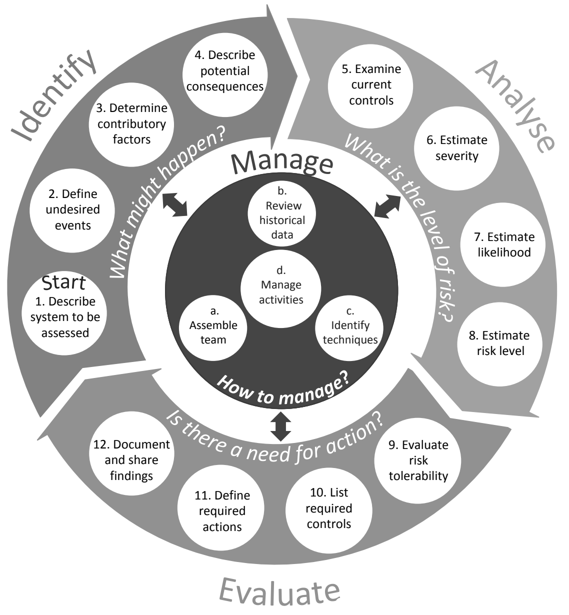

Glossary

Consequence:	Outcome of an **event**
Contributory factors:	Factors that contribute to the occurrence of an **event**
Control:	A measure that modified the **risk**[1]
Event:	Occurrence of a particular set of circumstances[1]
Likelihood:	Chance of a **risk** occurring[1]
Risk:	A potential undesired **event** that has effect(s) on objectives
Risk level:	Magnitude of a **risk** expressed by combining **consequences** and their **likelihood** [1]
Tolerability:	The degree of acceptability of a **risk**
Severity:	Seriousness of a **consequence**
System:	A combination of interacting elements organised to achieve stated purpose(s) [2]

1. ISO 73:2009, 2009. *Risk management- vocabulary*
2. ISO/IEC 15288, 2008. *Systems and software engineering- system life cycle processes*

Describe system to be assessed 01

Identify | Analyse | Evaluate | Manage

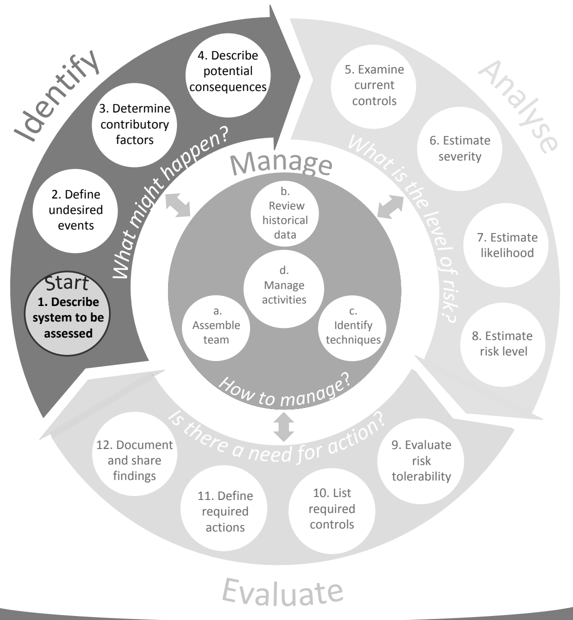

What is being assessed and how does the system work?

Describe system to be assessed 01

The following factors should be considered to describe the system to be assessed:

- Assessment aim
  What does the assessment aim to achieve?
- System elements
  What are the parts of the system?
- Interactions of the system elements
  What is the relationship between the system elements?
- System boundary
  What is the scope of the system?
- System context
  What is around the system to be assessed?

Since the following steps will be built on this step, it is essential to describe the system well.

What is being assessed and how does the system work?

Define undesired events 02

Identify | Analyse | Evaluate | Manage

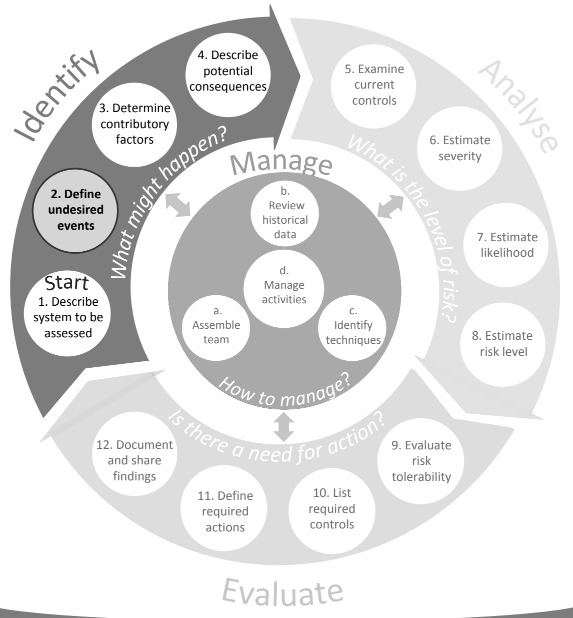

What could go wrong?

Define undesired events 02

The following should be considered when defining undesired events:

- System description (i.e. aim, elements, interactions, boundary and context)
- Extreme cases (e.g. fire)

Undesired events can be related to:

- Clinical practice (e.g. delayed discharge)
- Organisation (e.g. bed shortage)
- Health and safety (e.g. fire)
- Information (e.g. breach of confidentiality)

What could go wrong?

Determine contributory factors 03

Identify | Analyse | Evaluate | Manage

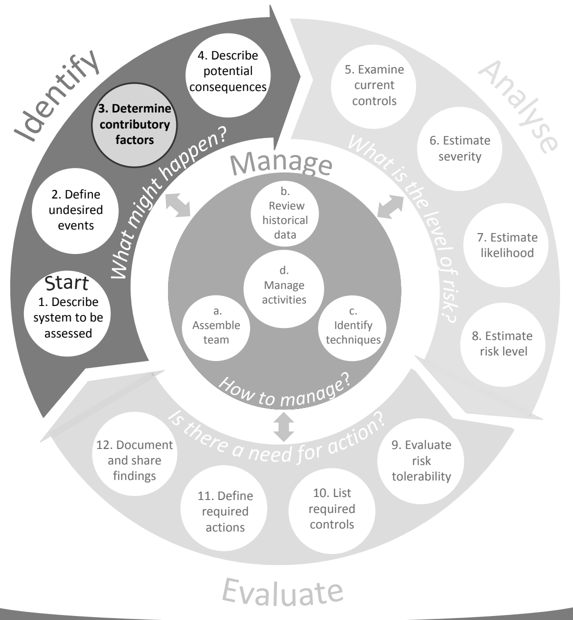

What could contribute to the occurrence of undesired events?

Determine contributory factors 03

Patient	Clinical condition, physical factors, social factors and psychological factors
Staff	Physical factors, psychological factors, social factors, cognitive factors, and skills and knowledge
Task	Unfamiliar task, difficult task and monotonous task
Communication	Poor verbal and written communication, lack of feedback between all stakeholders, and lack of information provided
Equipment	Poor design, equipment not working, and inadequate maintenance
Control actions	No actions, unsafe actions, actions too late, too early or out of sequence and actions stopped too soon or applied too long
Organisational	Organisational structure, policies, procedures and protocols, staffing/workload factors, training and safety culture
Environmental	Physical environment, external environment including external authorities and suppliers

What could contribute to the occurrence of undesired events?

Describe potential consequences 04

Identify | Analyse | Evaluate | Manage

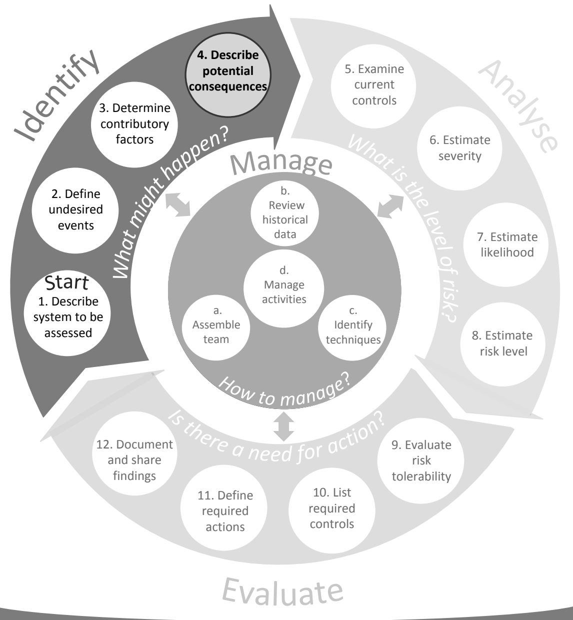

What are the potential consequences of the undesired events?

Describe potential consequences 04

The following should be considered when describing potential consequences:

- Impacts on people (e.g. harm and delayed treatment)
- Impacts on organisation (e.g. claims and complaints, staffing, financial loss and reputation)
- Impacts on environment (e.g. hospital waste and complaints from local residents)
- Immediate effects
- Knock-on effects

What are the potential consequences of the undesired events?

Examine current controls 05

Identify | Analyse | Evaluate | Manage

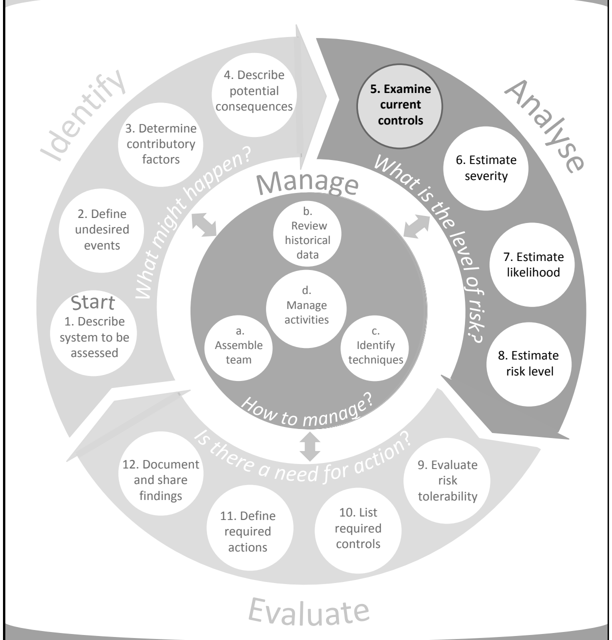

What are the current controls and how effective are they?

Examine current controls 05

The following types of controls can be considered when examining current controls:

- Controls to prevent undesired events
- Controls to detect undesired events
- Controls to reduce the severity of the consequences

The effectiveness of the current controls can be categorised as:

- Effective
- Neutral
- Ineffective

What are the current controls and how effective are they?

Estimate severity 06

Identify | Analyse | Evaluate | Manage

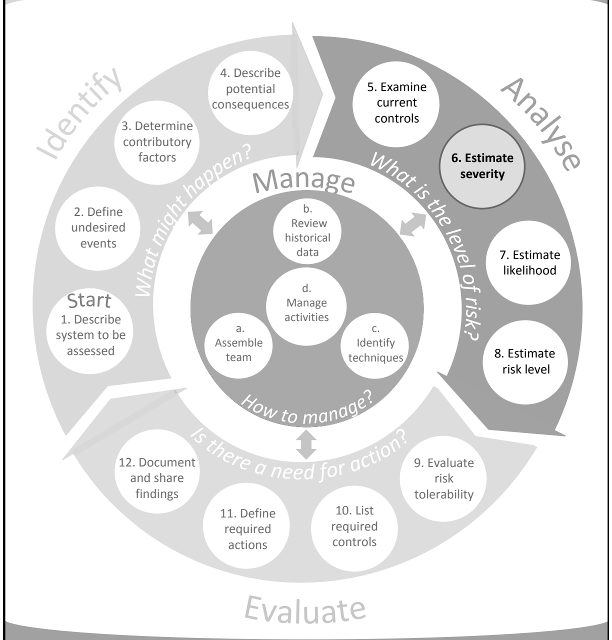

How severe are the described consequences?

Estimate severity 06

Severity can be estimated through the use of:

- A rating (see below)
- Consequence descriptions for each impact area (e.g. harm, staffing and reputation)

Score	Rating	Descriptions for harm
1	Negligible	Minimal injury requiring no intervention
2	Minor	Minor injury requiring intervention
3	Moderate	Moderate injury increasing the length of hospital stay
4	Major	Major injury leading to incapacity
5	Catastrophic	Death

Source: NPSA, 2008. A risk matrix for risk managers

If a risk might result in different severity of consequences on the same consequence category (e.g. harm), the most worst-credible can be determined.

How severe are the described consequences?

Estimate likelihood 07

Identify | Analyse | Evaluate | Manage

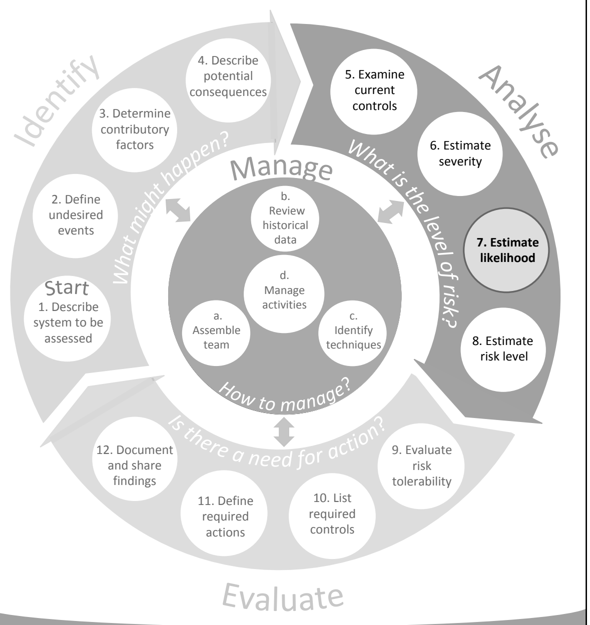

What is the likelihood of occurrence of the consequences?

Estimate likelihood 07

Likelihood of occurrence can be estimated through the use of:

- A rating (see below)
- Frequency descriptions to be used for continuous operations
- Probability descriptions to be used for one-off projects

Score	Rating	Frequency Descriptions	Probability Descriptions
1	Rare	Not expected to occur for years	<0.1 %
2	Unlikely	At least annually	0.1- 1 %
3	Possible	At least monthly	1- 10 %
4	Likely	At least weekly	10- 50 %
5	Almost certain	At least daily	>50 %

Source: NPSA, 2008. A risk matrix for risk managers

What is the likelihood of occurrence of the consequences?

Estimate risk level 08

Identify | Analyse | Evaluate | Manage

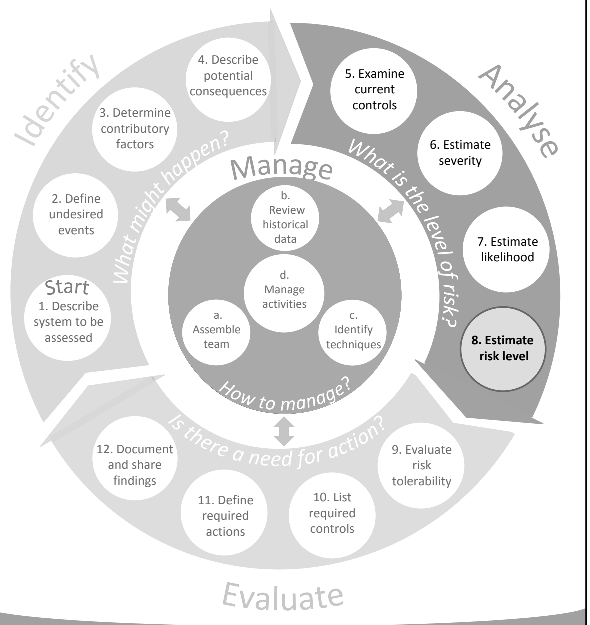

What is the level of risk?

Estimate risk level 08

Risk level is estimated by:

- Combining the likelihood and consequence of a risk

Risk levels can be categorised as:

- Low (L)
- Medium (M)
- High (H)

Likelihood \ Severity	1 Negligible	2 Minor	3 Moderate	4 Major	5 Catastrophic
5 Almost certain	M	M	H	H	H
4 Likely	L	M	M	H	H
3 Possible	L	L	M	M	H
2 Unlikely	L	L	L	M	M
1 Rare	L	L	L	L	M

What is the level of risk?

Evaluate risk tolerability 09

Identify | Analyse | **Evaluate** | Manage

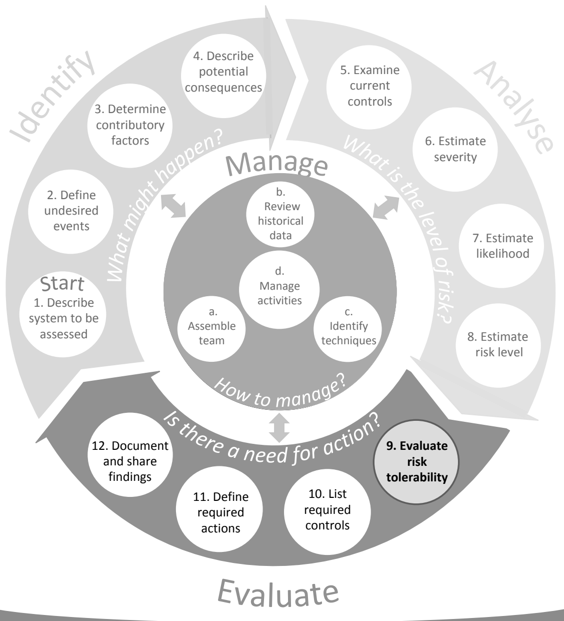

How tolerable is the risk?

Evaluate risk tolerability 09

The following should be considered when deciding on the tolerability of a risk:

- Risk level
  - Low risks: generally tolerable
  - Medium risks: generally undesirable
  - High risks: generally intolerable
- Written rules (e.g. standards, policies and legal requirements)
- Potential benefits of taking the risk

How tolerable is the risk?

List required controls 10

Identify | Analyse | **Evaluate** | Manage

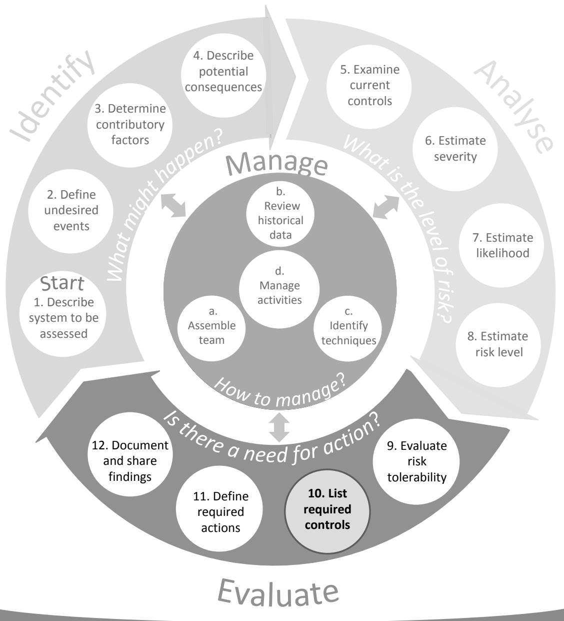

What new controls are required to modify the risk?

List required controls 10

The following should be considered to list new controls:

- Existing ineffective controls
- Contributory factors
- Controls to prevent undesired events
- Controls to detect undesired events
- Controls to reduce the severity of consequences

Be aware that new controls can raise new risks into the system, and some risks might not be eliminated.

What new controls are required to modify the risk?

Define required actions 11

Identify | Analyse | Evaluate | Manage

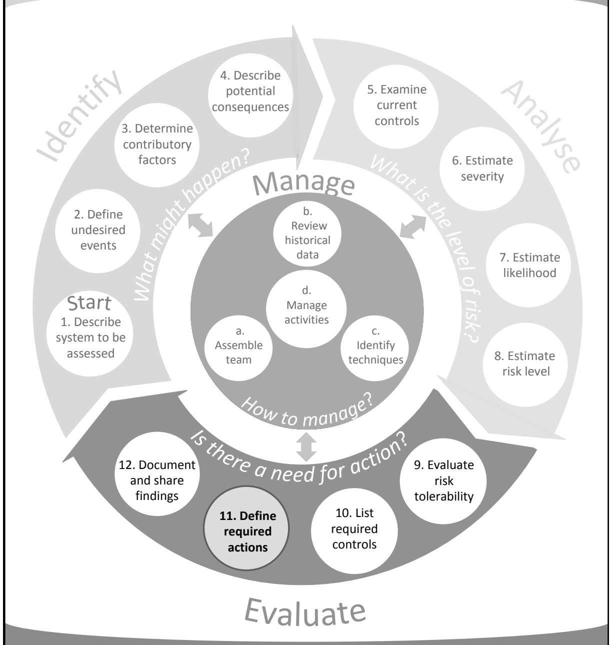

What actions are required to implement the new controls?

Define required actions 11

Required actions involve:

- Creating a list of actions in relation to the new controls
- Action prioritisation by considering the criticality of the risks (e.g. risk level, speed of a risk to manifest itself and its detectability, organisational objectives, rules and legal requirements)
- Management responsibility for these actions (e.g. ward/departmental level for low risks)
- Review frequency

Recommended actions should be 'SMART':

- Specific
- Measureable
- Achievable
- Realistic
- Timely

What actions are required to implement the new controls?

Document and share findings 12

Identify | Analyse | Evaluate | Manage

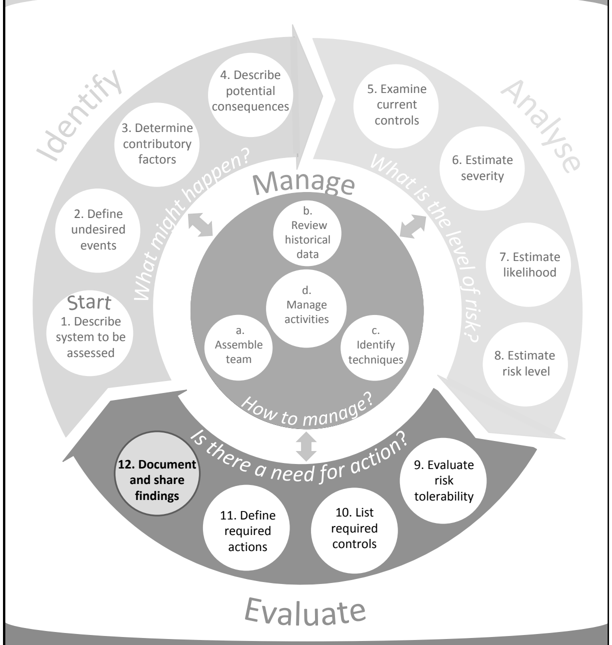

What are the findings and what lessons are learnt?

Document and share findings 12

Documentation of a risk assessment can include:

- Description of the system to be assessed
- Limitations and assumptions made in the assessment
- Assessment methodology
- Risk assessment findings and results
- Discussion of the results
- References

Findings can be shared with others through:

- Reports
- Safety alerts
- Risk newsletters
- Dashboards
- Communication with others

What are the findings and what lessons are learnt?

Assemble team a

Identify | Analyse | Evaluate | **Manage**

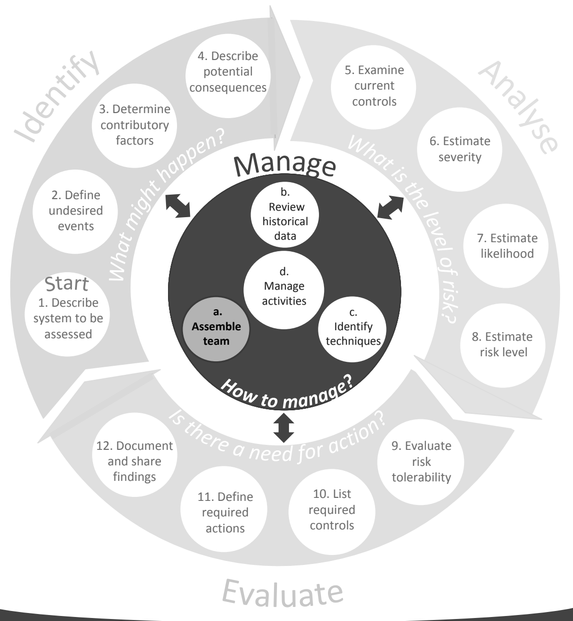

Who should be in the assessment team?

Assemble team a

An ideal team should involve at least:

- A facilitator who has experience in risk assessment
- A multidisciplinary group of experts in the system to be assessed

It might also be helpful to have:

- Somebody who is not directly involved in the system to be assessed

Be aware if a team assessment is not appropriate, peer reviewing is recommended to minimise subjectivity of the assessment.

Who should be in the assessment team?

Review historical data b

Identify | Analyse | Evaluate | **Manage**

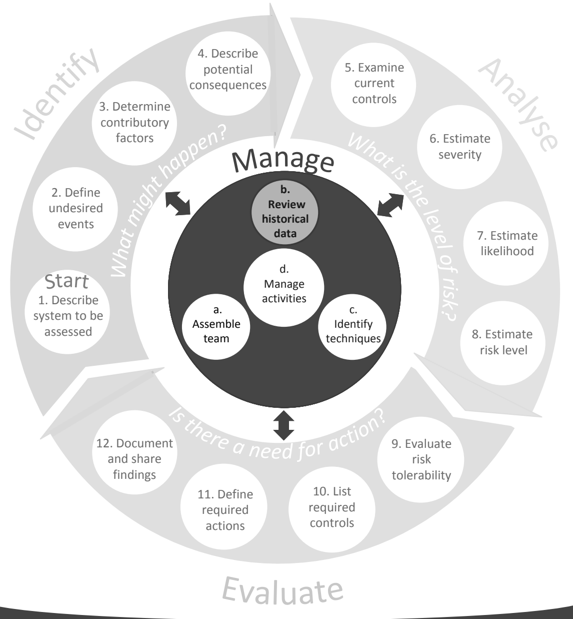

What can be learnt from historical data?

Review historical data b

The following documents and data can be used to provide insides from existing data:

- Incident reports
- Patient complaints and claims
- Registered risks
- Quality and performance reports
- Safety alerts
- Audit reports
- Reports from external authorities
- Academic literature

What can be learnt from historical data?

Identify techniques

c

Identify | Analyse | Evaluate | Manage

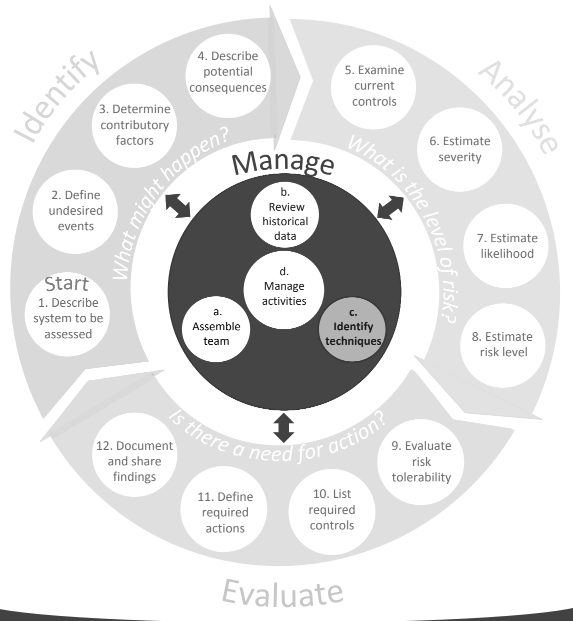

Which techniques should be used?

Identify techniques

c

Techniques to support risk assessment include:

- *System diagrams* or *flow charts* to describe the system to be assessed
- *Peer review* and *team discussion* to improve judgement
- *Brainstorming, structured 'what-If' (SWIFT)* and the *Delphi technique* to identify all risks
- *Bow-tie analysis* to display the pathway of an event from its contributory factors to potential consequences, and to examine current controls
- *Failure mode and effects analysis (FMEA)* to identify the way failures could occur and the way they could be treated
- *Risk matrices* to estimate the risk level, to determine risk tolerability and to allocate resources
- Specific risk assessment techniques (e.g. patient falls and moving and handling risk assessment forms)

Which techniques should be used?

Manage activities

d

Identify | Analyse | Evaluate | Manage

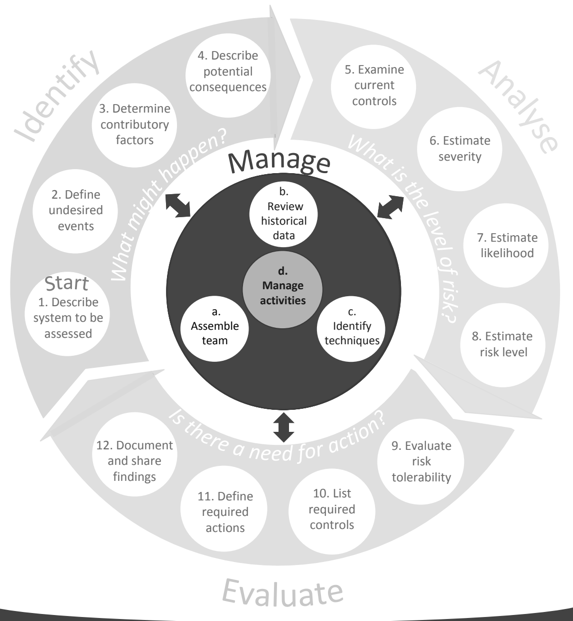

How should all risk assessment activities be managed?

Manage activities

d

It is essential to determine the following factors when managing the risk assessment process:

- Coordination of all risk assessment activities
- Communication and consultation with all stakeholders of the assessment at all times
- Iterating through all steps of the risk assessment framework
- Monitoring and reviewing assessed risks on a regular basis as well as when there is a change in the system
- Tailoring the framework to fit assessment needs

How should all risk assessment activities be managed?

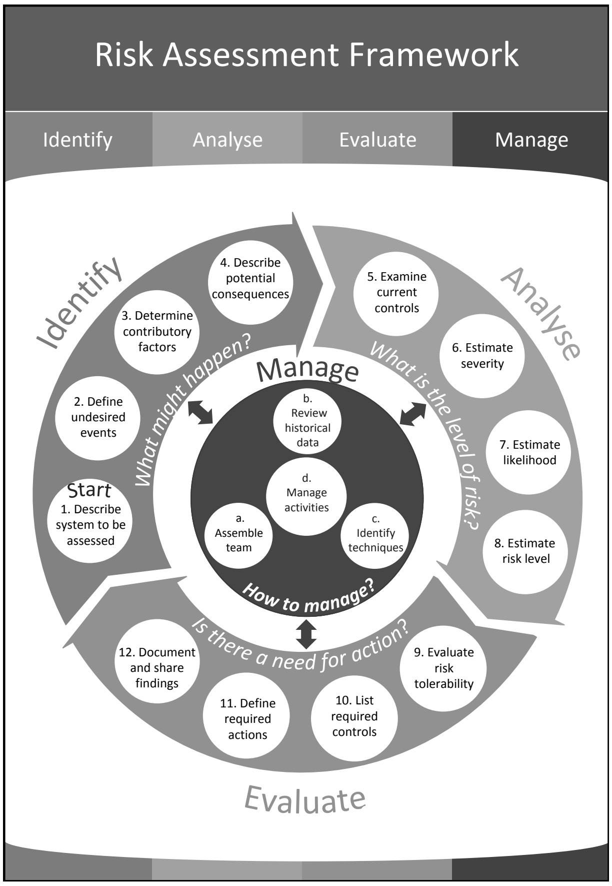

Risk Assessment Form

Assessor:	Ward/Department:	Date assessed:	Assessment no:

1.Describe system to be assessed
(aim, elements, interactions, boundary, context)

2.Define undesired events	3.Determine contributory factors	4.Describe potential consequences	5.Examine current controls	6.Estimate Severity	7.Estimate likelihood	8. Estimate risk level	9.Evaluate risk tolerability	10.List required controls	11.Define required actions
